# Characterization of fatty acid desaturase gene family in *Glycine max* and their expression patterns in seeds after *Fusarium fujikuroi* infection

**DOI:** 10.3389/fpls.2025.1540003

**Published:** 2025-02-25

**Authors:** Xinyuan Li, Maira Munir, Weiying Zeng, Zudong Sun, Xiaoli Chang, Wenyu Yang

**Affiliations:** ^1^ College of Agronomy and Sichuan Engineering Research Center for Crop Strip Intercropping System, Sichuan Agricultural University, Chengdu, Sichuan, China; ^2^ Institute of Economic Crops, Guangxi Academy of Agricultural Science, Nanning, Guangxi, China

**Keywords:** *Glycine max*, fatty acid desaturases, phylogenetic analysis, gene expression, *Fusarium fujikuroi*, seed decay

## Abstract

**Background:**

The family of membrane-bound fatty acid desaturase (*FAD*) genes play a vital role in plant growth, development, and stress responses. The seed-borne pathogen *Fusarium fujikuroi* causes seed decay disease during pre-harvest and post-harvest stages of soybean, leading to a significant reduction in yield and quality. Therefore, it is very meaningful to characterize the diversity and function of the *GmFAD* gene family in soybean and to elucidate their roles in seed resistance to *F. fujikuroi.*

**Results:**

In this study, 30 full-length *GmFAD* genes were identified from the soybean genome. A range of analysis was conducted to characterize gene and protein structures, chromosomal locations, conserved motif and conserved structural domains, and results showed that *GmFAD* genes were clustered into seven subfamilies (*FAB2*, *ADS*, *SLD*, *DES*, *FAD6*, *FAD2*, *FAD3/7/8*), which is also supported by phylogenetic analysis. The diversity and expansion of the *GmFAD* gene family were mainly caused by segmental duplication, and their encoding proteins were observed to locate in chloroplast or endoplasmic reticulum. The promoters of *GmFAD* genes contained a set of cis-acting elements in response to plant hormone, defense and stress, light, and plant growth and development, indicating these genes have the complex expression regulation and diverse functions. Gene ontology (GO) and KEGG enrichment pathway analyses showed that *GmFAD* genes were closely related to the biosynthesis and metabolism of lipid and unsaturated fatty acids (UFAs). In addition, the expression of *GmFADs* was significantly changed in soybean seeds when challenged by the seed decay pathogen *F. fujikuroi*. Specifically, *GmFAB2.1/2.2*, *GmFAD3.3/3-2B/7-1//8-2*, and *GmFAD2.3/2.5* genes displayed distinct temporal expression patterns in the resistant ND25 and susceptible CX12, highlighting their potential roles in soybean resistance against *F. fujikuroi* infection.

**Conclusion:**

Our findings contribute to a deeper understanding of the *GmFAD* gene family and their intricate roles in soybean resistance against the seed-borne pathogen *F. fujikuroi*. Moreover, several distinct genes provide valuable candidates for further application in soybean resistant breeding.

## Background

Unsaturated fatty acids (UFAs) have been increasingly recognized as significant components in the plant defense against a range of biotic and abiotic stresses (He et al., 2018). Fatty acid desaturases (FADs) are key enzymes in the biosynthesis of UFAs, catalyzing the insertion of double bonds at specific sites in fatty acid chains, thereby enhancing the fluidity and structural integrity of cell membranes (Dong et al., 2016; Feng et al., 2017). Generally, plant FADs can be categorized into soluble desaturases and membrane-bound desaturases based on their solubility (Nayeri and Yarizade, 2014). Among them, stearoyl-ACP desaturase, namely FAB2, known as the typical soluble FAD located in the plastid matrix, is responsible for introducing a double bond at the Δ9 position, and facilitating the conversion of stearic acid to oleic acid (Ha et al., 2010; Xue et al., 2018). Membrane-bound FADs are further divided into four distinct subfamilies based on their functions, including omega-6 desaturases (ω6, FAD2 and FAD6), omega-3 desaturases (ω3, FAD3, FAD7 and FAD8), FAD4, and DES/ADS/SLD (Hashimoto et al., 2008; Lou et al., 2014; Saini and Kumar, 2019). Generally, FADs in the same subfamily have the highly conserved amino acid sequences (Hashimoto et al., 2008). To date, FAD genes have been characterized in many plants, such as soybean (*Glycine max*) (Chi et al., 2011), tobacco (*Nicotiana tabacum*) (Sayanova et al., 1997), banana (Cheng et al., 2022), corn (*Zea mays*) (Mikkilineni and Rocheford, 2003), rice (*Oryza sativa*) (Wang et al., 2006), etc.

Numerous studies have demonstrated that *FADs* play crucial roles in plant stress tolerance, such as high and low temperatures, drought, salinity, and heavy metal exposure (Xue et al., 2018). The *MaFADs* expression in banana are significantly activated in response to high and low temperature (Cheng et al., 2022). Similarly, a significant up-regulation of *CsSLD3* and *CsSLD4* is also observed under cold stress in *Camellia sinensis* (Jin et al., 2024). Overexpression of antisense *AtFAD2* results in a decrease tolerance to drought and salt stresses in *Arabidopsis* (Im et al., 2002), when the transcript of the soybean homologous *GmFAD2-2C* was accumulated to increase in pods grown in cool conditions rather than those in warmer conditions (Li et al., 2007). *AtFAD3* or *AtFAD8* expressed in transgenic tobacco enhanced the resistance to drought and osmotic stresses (Zhang et al., 2005). On the other hand, since fatty acids as the main source of organic carbon can be delivered to the fungi by the host plants, thus *FADs* as pivotal agents in plant lipid metabolism also modulate the plant-pathogen interaction (Jiang et al., 2017; Luginbuehl et al., 2017). Researches have demonstrated that triene fatty acids originating from chloroplasts are involved in host resistance at the infection initial stage of pathogen (Chandra-Shekara et al., 2007). Transient silencing of the *FAD2* homologous gene in wheat (*Triticum Aestivum*) increased its susceptibility to powdery mildew (Jiang et al., 2009). Conversely, the inhibition of *OsFAD7* and *OsFAD8* have been found to enhance the transgenic rice resistance against *Magnaporthe grisea* (Yara et al., 2007). In addition, the *SSI2* gene encoding a stearoyl-acyl carrier protein-desaturase (SACPD) also participates in the pathogen resistance, and knockdown of *OsSSI2* markedly increased accumulation of endogenous free salicylic acid (SA) and enhanced rice resistance to the fungus *Magnaporthe grisea* and bacterium *Xanthomonas oryzae* pv. *oryzae* (Li et al., 2011). Similarly, overexpression of the *Arabidopsis ssi2* mutant *TaSSI2* restored its resistance to powdery mildew fungi (Song et al., 2013). In soybean, the fatty acid composition in soybean tissues is often responsive to pathogen attack (Upchurch, 2008), and fatty acids and fatty acid-derived compounds act as signals of defense gene expression (Upchurch, 2008). Evidence suggests that the level of stearic acid and oleic acid are critical for defense against pathogens in soybean as they have been shown to be in *Arabidopsis* (Kachroo and Kachroo, 2009). Moreover, the oleate and linoleate content of soybean seeds appears to influence the course of seed colonization by *Cerospora kikuchii* and *Diaporthe phaseolorum* (Xue et al., 2008). Silencing of *GmSACPDs* confers soybean resistance to *Pseudomonas syringae* pv. *glycinea* (Kachroo et al., 2008). Thus, *FADs* as crucial regulatory components are capable of reacting to and being linked with various stress-induced damages in plants.

Soybean (*Glycine max*) is one of the largest oilseed crop worldwide and rich in high-quality vegetable protein and unsaturated fatty acids. However, soybean seed quality and yield are often affected by various seed-borne diseases (Wrather et al., 2003). Specially, seed decay emerges as one of the most damaging seed-borne diseases during the pre- and post-harvest stages of soybean, and this disease often leads to substantial yield losses, poor seed quality and nutrients, and reduced seed germination and vigor (Chang et al., 2020a; Xu et al., 2023). Some infected seeds can even become important carriers of diseases, facilitating the spread of these pathogens over extensive distances (Lv and Sun, 2007). The necrotrophic fungus *Fusarium fujikuroi* has previously been reported to cause soybean root rot (Chang et al., 2020b), and it was also identified as the causal agents of pod blight and seed decay (Suga et al., 2018; Chang et al., 2022). The fungus is capable of producing a variety of secondary metabolites, such as fumonisins and gibberellins, which threaten the health of humans and livestock (Zhang et al., 2021). However, there is little information on *F. fujikuroi-*induced seed responses in soybean. Recently, our study showed that the soybean cultivar CX12 exhibited susceptibility to *F. fujikuroi*, whereas the cultivar Nandou25 had high resistance to *F. fujikuroi*. Given the important role of *FAD* genes in enhancing plant resistance against various diseases, it become meaningful to explore the contribution of *GmFAD* to soybean seed decay.

To date, the *GmFAD* gene family has been reported in *Glycine max* through genomic and transcriptomic analyses (Chen et al., 2016; Liu et al., 2017). A total of 75 *FAD* genes have already reported from the genomes of different soybean species, with 23 *FAD* genes found in *Glycine max* var. Williams 82 (Chen et al., 2016). However, the diversity and function of *GmFAD* genes in soybean remain largely uncovered. This study aimed to analyze the functions of the soybean *FAD* gene and clarify the roles of *GmFAD* in soybean resistance to the seed-borne *F. fujikuroi*. By performing a genome-wide analysis using the latest genomic data from *Glycine max* var. Williams 82, we systematically characterized and functionally annotated the *GmFAD* gene family according to their physicochemical properties, gene structures, and promoter motifs. Furthermore, we analyzed the expression profiles of representative *FAD* genes using qRT-PCR during *F. fujikuroi* infection in soybean seeds. This study aids in identifying candidate genes that may improve *Fusarium* resistance in soybean.

## Results

### Identification of *GmFAD* genes in soybean

Full-length genes of 30 fatty acid desaturase (*GmFADs*) were predicted from the genome of *Glycine max* var. Williams 82 (*Glycine max Wm82.a4.v1*) using HMM search, and were listed in **Supplementary Table S1**. The identified proteins corresponding to the *GmFAD* gene family had an amino acid sequence length from 235 aa (*GmFAD6.2*) to 453 aa (*GmFAD7-2*). The relative molecular mass of these proteins varied between 26,800.2 Da (*GmFAD6.2*) and 51,550.41 Da (*GmSLD1.2*). The isoelectric points (pI) of the proteins were distributed within a range of 5.94 (*GmFAB2.1*) to 9.51 (*GmFAD5.1*), with the majority possessing pI values above 7. The lipolysis index values ranged from 79.26 (*GmSACPD*) to 94.62 (*GmSLD1.4*). The instability coefficients of proteins were found to vary between 30.72 and 48.67, with *GmFAD2.3* protein being the most stable and *GmFAD6.1* protein the least stable among these members identified. Out of the 30 family members, 21 members were classified as hydrophilic proteins, whereas the others were hydrophobic (**Supplementary Table S2**).

The subcellular localization analysis predicted that proteins *GmFAD2*, *GmDES*, and *GmSLD* were localized on endoplasmic reticulum, whereas *GmFAD6* and *GmFAB2* proteins localized on chloroplasts. The members of *GmFAD5* and *GmFAD3* along with their isozymes *GmFAD7/FAD8* proteins were found in both endoplasmic reticulum and chloroplasts as detailed in **Supplementary Table S2**. These results suggest that the subcellular location diversity of these proteins could be related to multiple functions.

### Phylogenetic relationship analysis of GmFAD proteins

**Figure 1:**
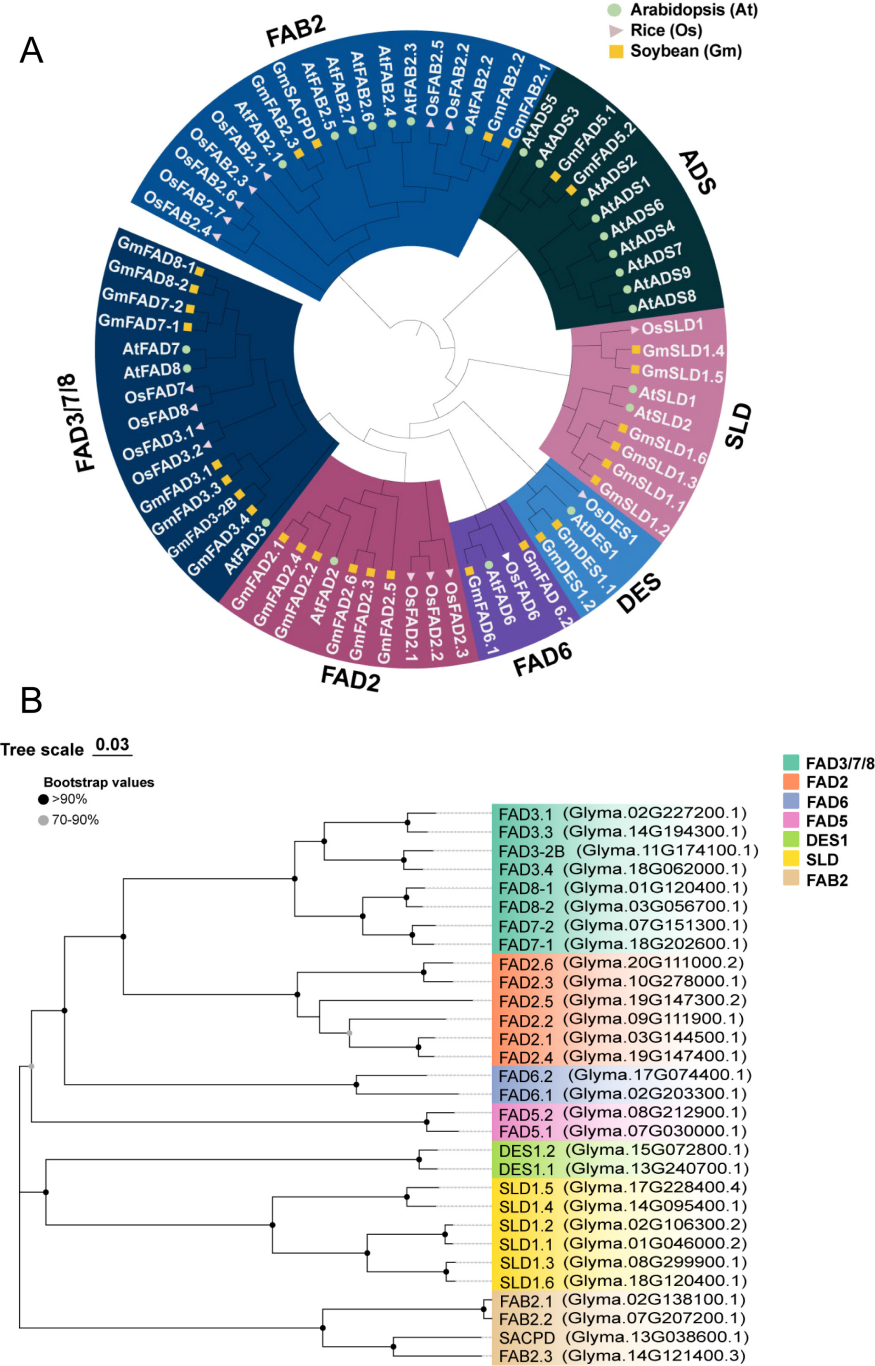
Phylogenetic analysis of FADs proteins. **(A)** Phylogenetic relationship of FAD proteins from *Arabidopsis* (*At*), rice (*Os*) and soybean (*Gm*). The amino acid sequences of GmFAD, AtFAD and OsFAD were compared using ClustalW and a phylogenetic tree was constructed using the neighbor-joining (NJ) method with p-distance model by MEGA7.0. The bootstrap support values were calculated from 1000 replicates. FAD proteins are divided into seven subfamilies as FAD3/7/8, FAD2, FAD6, DES, ADS, SLD and FAB2, which are indicated by different colors. **(B)** Phylogenetic relationships of GmFAD protein in soybean.

To determine the evolutionary relationships of FAD proteins among *A. thaliana* (*At*), rice (*Os*), and soybean (*Gm*), a phylogenetic tree was constructed using the neighbor-joining (NJ) method with p-distance model using amino acid sequences of GmFADs (30), AtFADs (24), and OsFADs (18) (**Supplementary Table S3**). As depicted in **Figure 1A**, all FAD proteins were classified into two distinct clusters: soluble (FAB2) and membrane-bounding FAD proteins (ADS, SLD, DES, FAD6, FAD2, and FAD3/FAD7/8).

FAB2 subfamily represents the only soluble desaturase identified so far. In this study, both *A. thaliana* and rice were found to contain seven homologous proteins, respectively (**Figure 1A**). In contrast, it was observed that soybean possesses four FAB2, namely three GmFAB2 and one GmSACPD, and among them, two alleles GmFAB2.1 and GmFAB2.2 were clustered together, when GmFAB2.3 aligned with GmSACPD in a different branch (**Figure 1B**). Compared FAB members in three species, except for OsFAB2.1, AtFAB2.1 and OsFAB2.1, most FAB members of soybean and *A. thaliana* were closely clustered together but OsFAB in rice was located at the base position of this subfamily. This suggests that FAB2 in rice may have evolved earlier and could have been divided into monocotyledonous and dicotyledonous taxa.

As shown in **Figure 1A**, proteins encoding membrane-bound desaturases were categorized into six distinct subfamilies. Among them, the ADS subfamily was further classified into two groups, composed of nine ADS from *A. thaliana* along with their two homologous FAD5 from soybean. Interestingly, GmFAD5.1 and GmFAD5.2 were closely grouped with AtADS3 and AtADS5 as a small branch, indicating that GmFAD5 might evolved from AtADS. Furthermore, no members from rice were identified in the ADS subfamily, indicating that it might have undergone evolutionary loss in rice or were transmitted to dicotyledons (*A. thaliana* and soybean) following their divergence from the last common ancestor.

The SLD subfamily comprised six GmSLD, two AtSLD, and one OsSLD, all of which encode sphingolipid Δ7 desaturases. Except for OsSLD1 was grouped with both GmSLD1.4 and GmSLD1.5 as a separate branch, the SLD members from each plant species were distinctly clustered together, indicating a closer genetic relationship among them. The DES subfamily, which is responsible for sphingolipid Δ4 desaturases, had the members including OsDES1, AtDES1, GmDES1.1 and GmDES1.2, with OsDES1 in rice localized at the basal position of the subfamily. This suggests GmDES1 in soybean as well as AtDES1 in *A. thaliana* had more close relationship as the dicotyledonous plants, but they diverged from OsDES1 from the last common ancestor.

Both FAD6 and FAD2 subfamilies encode Δ12 desaturases but were differently localized in the subcellular organelles. In the plastidial FAD6 subfamily, GmFAD6.2 notably occupied at a basal position within this subfamily. In the microsomal FAD2 subfamily, three rice OsFAD2 were clustered together, while six GmFAD2 and one AtFAD2 grouped in another branch, indicating this family has diverged into monocotyledonous and dicotyledonous groups from the same ancestor. Additionally, in the FAD3/FAD7/FAD8 subfamily, eight FADs from soybean (four *GmFAD3*, two *GmFAD7* and two *GmFAD8*), three from *Arabidopsis*, and four from rice, were clustered together and encoded their corresponding microsomal or plastidial ω3 desaturases.

### Chromosomal location and gene duplication analysis of *GmFAD* genes

**Figure 2:**
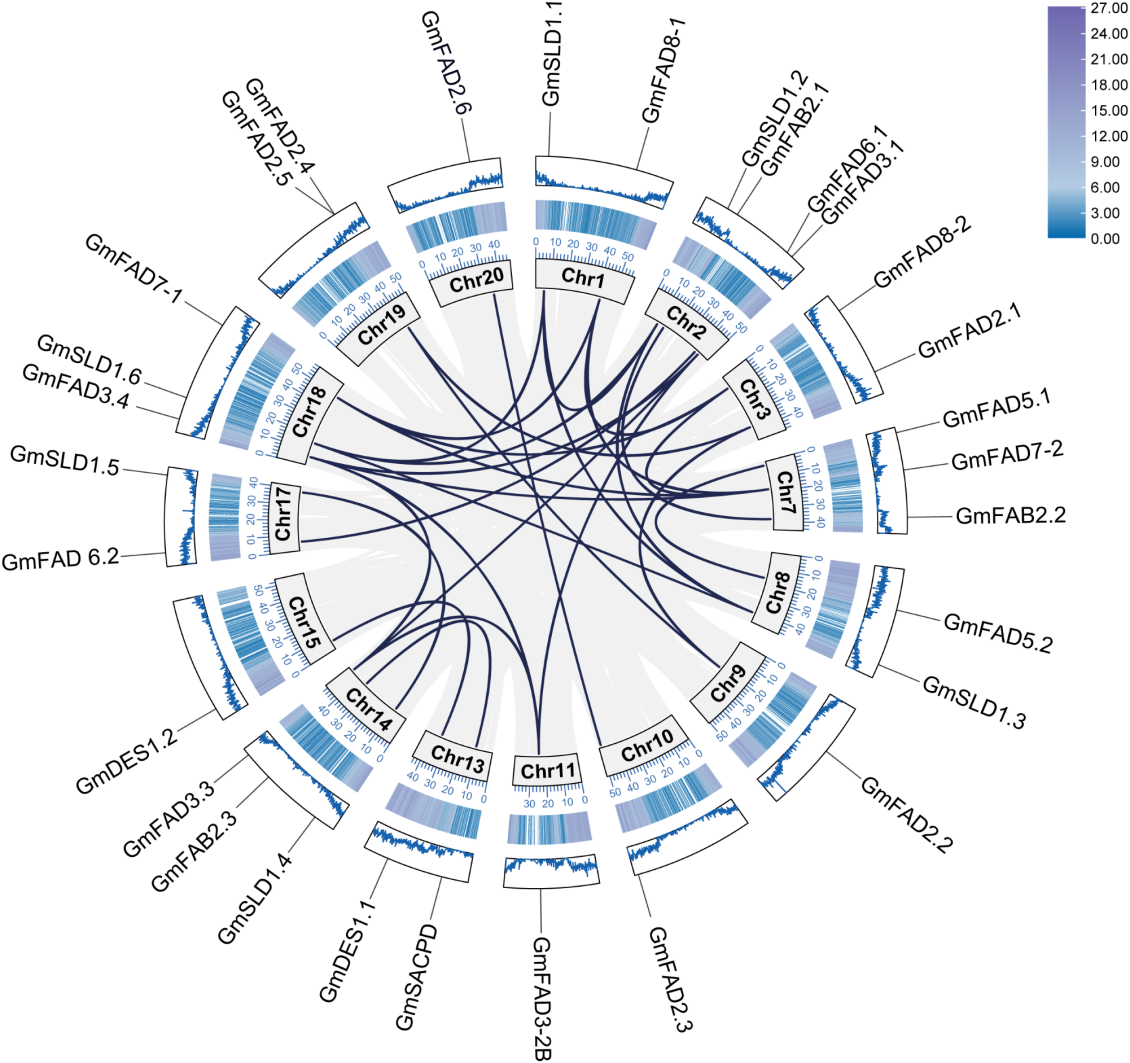
Covariance analysis of *GmFAD* gene family. Rectangles indicate chromosomes, and the location of the *GmFAD* genes on the chromosome is marked using the *GmFAD* name. Each gray and dark blue curves indicate all covariate gene pairs on the chromosome and gene duplication events of *GmFAD*, respectively.

As shown in **Figure 2**, different *GmFAD* genes were located in different soybean chromosomes. Except for the chromosomes 4, 5, 6, 12, and 16, the identified 30 *GmFAD* genes in soybean were found to distribute on the other 15 of the 20 soybean chromosomes. The maximum number of *GmFAD* genes were located on chromosome 2 as four genes. There were three genes located on the chromosomes 7, 14, and 18, respectively, while only one or two genes were located on other chromosomes. In addition, different gene members of each subfamily were mostly distributed in different chromosomes except that *GmFAD2.4* and *GmFAD2.5* was clustered in a small region of chromosome 19. This suggests that *GmFAD* genes are broadly dispersed throughout the soybean genome, and they might originate from diverse ancestors.

The diversity and expansion of gene families often arise from crosstalk and segmental duplication events, and gene duplication serves as a key mechanism for enhancing plant genetic diversity and the generation of novel genes. In this study, covariance analysis based on MCScanX was performed to investigate gene duplication events in the *GmFAD* gene family, and results showed that a large number of *GmFAD* genes had covariance between and within soybean chromosomes, and the most *GmFAD* genes with covariance were localized on the chromosome 19. As shown in **Table 1**, both tandem and segmental duplication can be observed in the *GmFAD* family. Except for *GmFAD2.4* and *GmFAD2.5* on the chromosome 19 were caused by tandem duplication, a total of 29 segmental duplication events were identified (**Table 1**). Among them, the ω-3 desaturase subfamily (*GmFAD3/GmFAD7/GmFAD8*) had the maximum duplicated gene pairs as 13 pairs followed by the *GmSLD* subfamily with seven pairs, while the ω-6 desaturase subfamilies (*GmFAD2* and *GmFAD6*) had five duplicated gene pairs. The subfamilies of *GmFAB2*, *GmDES* and *GmFAD5*(*ADS*) had less duplicated gene pairs than other subfamilies in our study. Thus, the role of segmental duplication events plays a prominent role in increasing the genetic diversity of soybean *GmFAD* gene families rather than tandem duplication.

**Table 1:** Ka/Ks analysis of the *GmFAD* gene pairs duplication.

Duplicated gene 1	Duplicated gene 2	Ka	Ks	Ka/Ks
*GmFAD8-1*	*GmFAD8.2*	0.02328	0.1421	0.16383
*GmFAD8-1*	*GmFAD7-2*	0.124333	0.709715	0.175188
*GmFAD8-1*	*GmFAD7-1*	0.120121	0.720123	0.166806
*GmFAD8.2*	*GmFAD7-2*	0.138577	0.755093	0.183523
*GmFAD8.2*	*GmFAD7-1*	0.135723	0.758853	0.178853
*GmFAD7-2*	*GmFAD7-1*	0.030214	0.096519	0.313034
*GmFAD7-2*	*GmFAD3.4*	0.219589	1.978483	0.110988
*GmFAD6.1*	*GmFAD6.2*	0.144787	0.23843	0.607254
*GmFAD5.1*	*GmFAD5.2*	0.033837	0.134296	0.25196
*GmFAD3.1*	*GmFAD3-2B*	0.145533	0.881057	0.16518
*GmFAD3.1*	*GmFAD3.3*	0.02335	0.130903	0.178379
*GmFAD3.1*	*GmFAD3.4*	0.134448	0.769057	0.174822
*GmFAD3-2B*	*GmFAD3.3*	0.145017	0.8803	0.164736
*GmFAD3-2B*	*GmFAD3.4*	0.023048	0.12135	0.189928
*GmFAD3.3*	*GmFAD3.4*	0.131717	0.79204	0.166302
*GmFAD2.1*	*GmFAD2.2*	0.134722	1.22811	0.109699
*GmFAD2.1*	*GmFAD2.5*	0.183761	0.500099	0.36745
*GmFAD2.2*	*GmFAD2.5*	0.271703	1.135618	0.239256
*GmFAD2.3*	*GmFAD2.6*	0.030866	0.123855	0.249212
*GmSLD1.1*	*GmSLD1.2*	0.007168	0.137411	0.052164
*GmSLD1.1*	*GmSLD1.3*	0.115137	0.964275	0.119403
*GmSLD1.1*	*GmSLD1.6*	0.113882	0.94864	0.120048
*GmSLD1.2*	*GmSLD1.3*	0.116353	0.883028	0.131766
*GmSLD1.2*	*GmSLD1.6*	0.115659	0.855715	0.135161
*GmSLD1.3*	*GmSLD1.6*	0.011562	0.216251	0.053465
*GmSLD1.4*	*GmSLD1.5*	0.03313	0.261431	0.126725
*GmFAB2.1*	*GmFAB2.2*	0.008886	0.200335	0.044354
*GmSACPD*	*GmFAB2.3*	0.117118	0.506882	0.231055
*GmDES1.1*	*GmDES1.2*	0.01621	0.105436	0.153738

The Ka/Ks value represents the ratio between synonymous substitutions (Ka) and non - synonymous substitutions (Ks) based on a whole genome analysis of gene duplication. A Ka/Ks ratio greater than 1 means the genes evolved under positive selection, a ratio of 1 indicates the genes evolved under neutral selection, while a Ka/Ks ratio less than 1 suggests negative purifying selection.

To assess the selection of duplicated *GmFAD* gene pairs, we calculated the substitution ratios between non-synonymous and synonymous (Ka/Ks) based on a whole genome analysis of gene duplication (**Table 1**). In soybean, most Ka/Ks ratios for the GmFAD gene replication pairs were found to be less than 1, indicating that these genes are relatively conserved during the evolutionary process and have been subjected to purifying selection pressure to maintain gene functions and species stability. However, three gene pairs, such as *GmFAD7-2/GmFAD3.4*, *GmFAD2.1/GmFAD2.2*, and *GmFAD2.2/GmFAD2.5*, exhibited a Ks value greater than 1, and it indicates that a positive selection pressure occurs to produce new protein functions aiming to promote gene evolution and the adaptive changes of species.

### Analysis of gene structures, conserved motifs and protein structures of *GmFAD* in soybean

**Figure 3:**
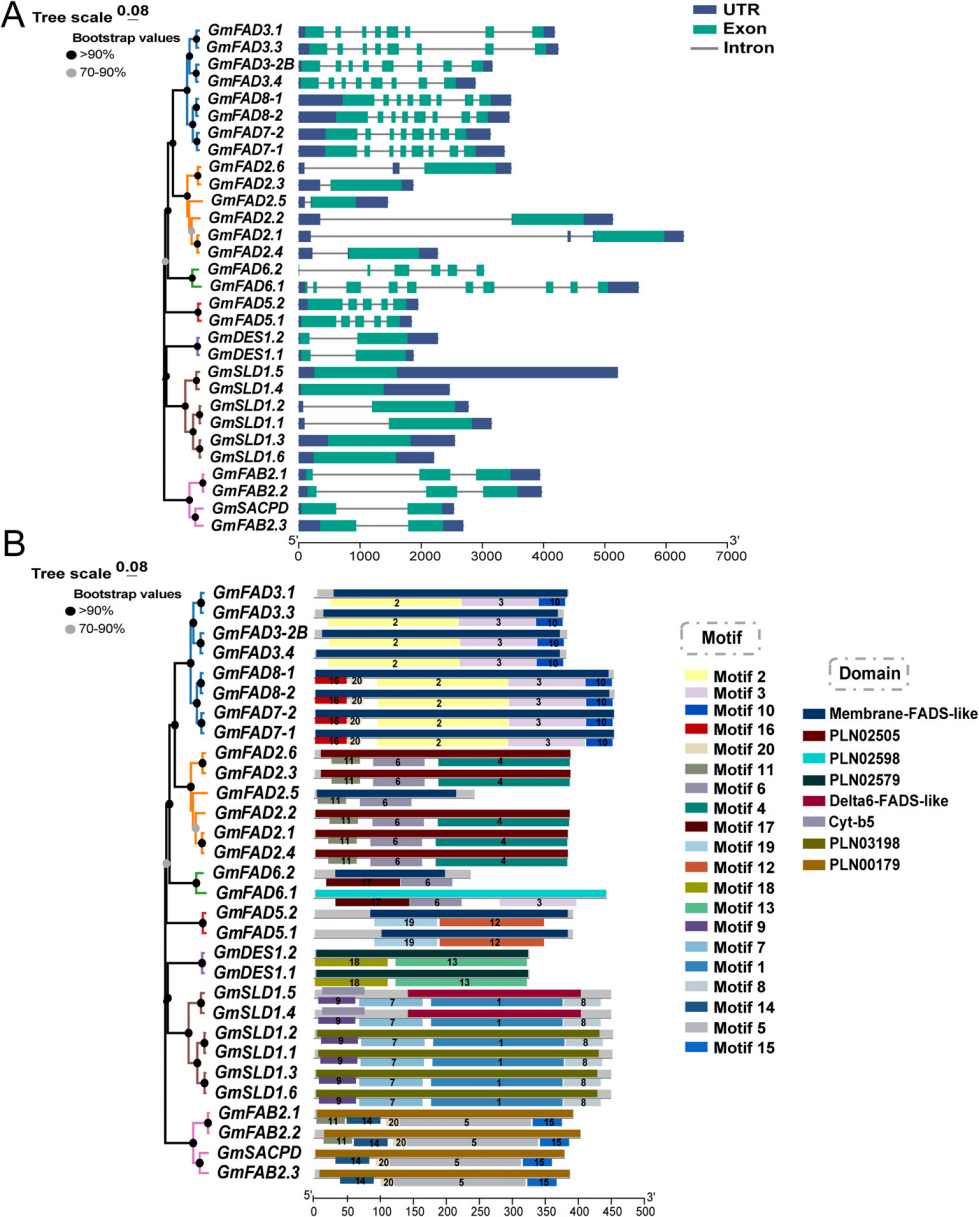
Analysis of gene structure, conserved motifs and conserved structural domain interactions in soybean GmFAD proteins. **(A)** Exons and introns of *GmFAD* genes. Green boxes and blue boxex represented exons and untranslated region (UTR), respectively. Grey lines meaned introns. **(B)** Conserved motifs of soybean *GmFAD* gene family and their interacted with conserved structural domains. Motifs 1-20 represent conserved motifs. Membrane-FADS-like PLN02505, PLN02598, PLN02579, Δ6-FADS-like, Cyt-b5, PLN03198 and PLN00179 belong to conserved structural domains.

Analysis of the exon/intron structures of 30 *GmFAD* genes identified in soybean (**Figure 3A**, **Supplementary Table S4**), showed that the gene structure of different *GmFAD* subfamily was generally variable. Both *GmFAD3/FAD7/FAD8* and *GmFAD6* gene subfamilies had more than 6 exons, especially *GmFAD6.1* possessed ten exons. *GmFAD5* subfamily had five exons as compared to *GmDES* and *GmFAB2* subfamilies which contained two to three exons. In addition, members in the *GmFAD* and *GmFAB* subfamilies had different number of exons, for example, *GmFAD6.2* and *GmFAD6.1* had four and ten exons, respectively. Interestingly, *GmFAD2* and *GmSLD* had the simplest structures with only one exon. Furthermore, most *GmFAD* genes within the same subfamily exhibited high similarity in their exon/intron patterns as compared to those in different subfamilies, thus the exon/intron distribution could provide strong supports for the phylogenetic classification of GmFAD proteins above.

Furthermore, the gene motifs were analyzed in this study, and total 20 motifs were identified in *GmFAD* genes (**Figure 3B**). Evaluation of motifs 1-20 using the Pfam database (http://pfam.xfam.org) showed that motifs 2, 3, 6, 10, 11, 12, 16, 19, and 20 corresponded to membrane-FADS-like superfamily structural domains, while other three protein conserved structural domains PLN02505, Δ6-FADS-like, and PLN02598 were also identified as the membrane-FADS-like superfamily. Motifs 2, 3, and 10 were observed in all members of the ω-3 subfamilies (*GmFAD3/GmFAD7/GmFAD8*), while two unique motifs 16 and 20 were found in the *GmFAD7* and *GmFAD8* subfamilies. All the *GmFAD2* subfamily members contained motifs 4, 6, and 11 except that *GmFAD2.5* lacked motif 4. Motif 17 was present only in the *GmFAD6* subfamily, but this motif is not functional. In the *GmFAB2* subfamily, motifs 5, 14, 15 and 20 corresponded to the acyl-[acyl-carrier protein] desaturase structural domain (PLN00179), whereas Motif 11 was a domain of unknown function (with PLN00179 domain) and only existed in the branch of *GmFAB2.3* and *GmSACPD* as compared to *GmFAB2.1* and *GmFAB2.2*. The members of *GmFAD5*(ADS), *GmDES*, and *GmSLD* subfamilies comprised two, two, and four motifs, respectively. Notably, motif 9, as a component of the Cyt-b5 domain, was uniquely identified in the *GmSLD* subfamily. In short, members with similar conserved motifs and gene structures clustered together in the *GmFAD* gene family. The motif distribution patterns of each subfamily are similar, whereas differences between subfamilies may be related to subfamily functional convergence.

**Figure 4:**
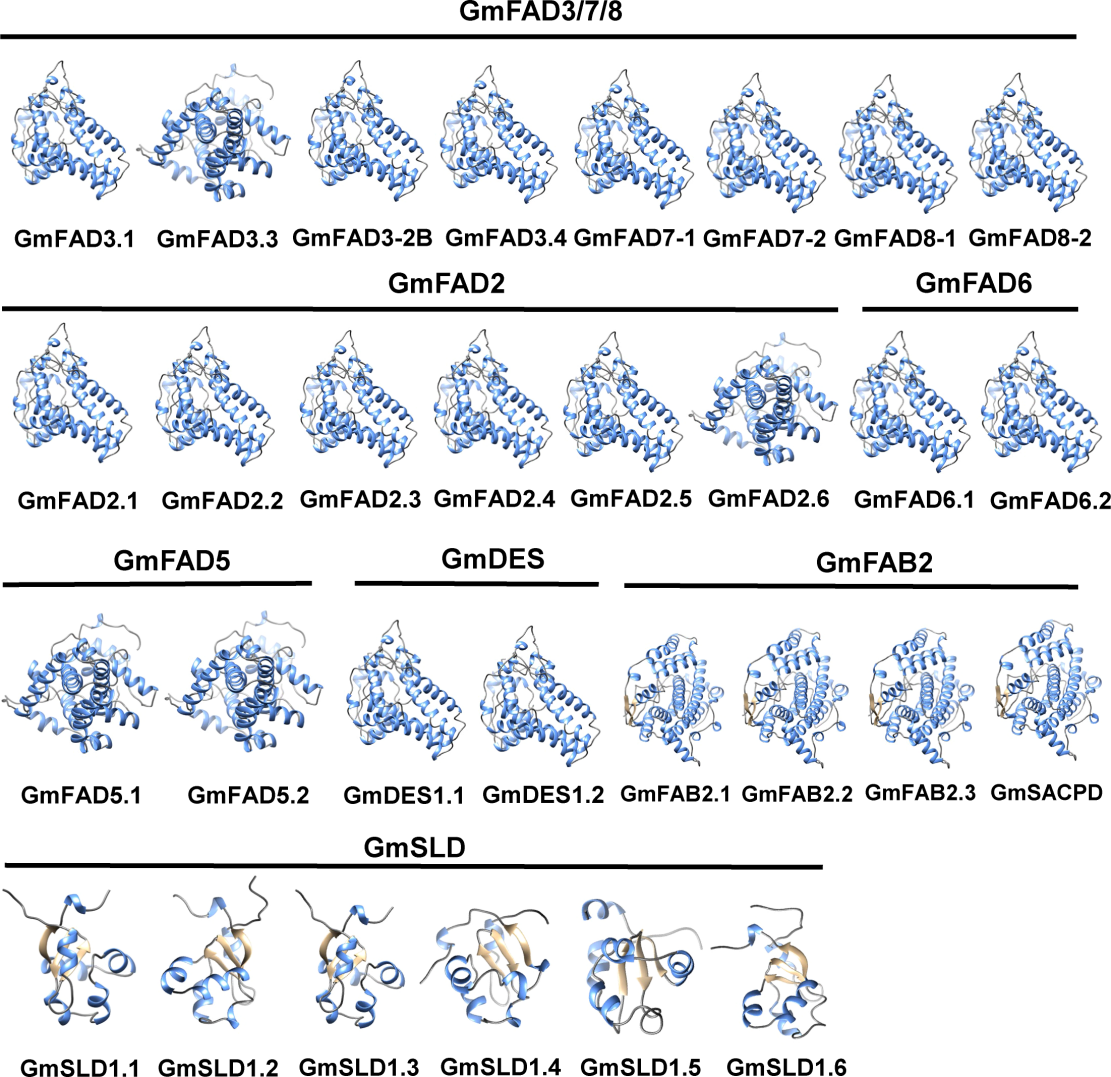
Three-dimensional conformation prediction of soybean GmFAD proteins. The coil, α-helix, and strand were represented in green, gray and yellow, respectively.

To elucidate the three-dimensional conformation of the GmFAD proteins, homology modeling method was adopted and homology modeling was performed using Swiss-Model online software. As shown in **Figure 4**, GmFAD primarily consisted of α-helixs, irregular coils, and β-turns, and proteins clustered into the same clade exhibited analogous 3-dimensional (3D) structures. GmFAD3.3 within the ω-3 desaturase subfamilies and GmFAD2.6 within the ω-6 desaturase subfamilies exhibited distinct characteristics compared to GmFAD proteins from other subfamilies, indicating that there exists a balance between conservation and divergence within the GmFAD proteins.

### Analysis of cis-acting elements in *GmFAD* gene promoter

**Figure 5:**
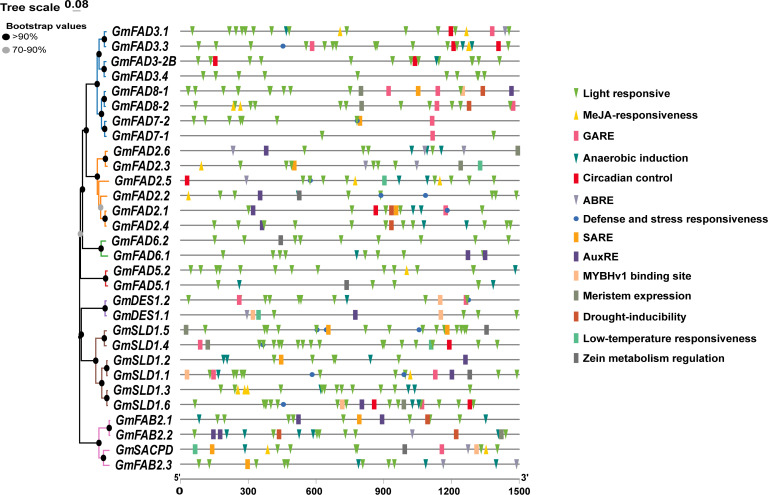
Map of cis-acting elements of the *GmFAD* gene promoter. Promoter sequences (−1500 bp) of *GmFAD* genes are speculated on PlantCARE. The upstream length to the translation start site can be deduced on the basis of the scale at the bottom.

Promoters located in the upstream regions of genes are crucial in gene expression regulation involving in plant growth and development as well as environmental adaptation. To understand the roles of *GmFAD* genes and the precise regulation of gene expression, cis-acting elements within the promoter regions were analyzed (**Figure 5**, **Supplementary Table S5**). The results showed that the promoter region of the *GmFAD* genes contained three distinct types of cis-elements. The first type of cis-elements was related to plant growth and development, including light responsiveness, circadian control, the differentiation of fenestrated chloroplasts, and cell cycle regulation. The second type was responsible for stress responses, including those triggered by methyl jasmonate (MeJA), abscisic acid (ABA), salicylic acid (SA), GA, growth hormone, low-temperature, drought, defense and stress responsiveness. The third category is associated with specific biological processes, such as those involved in endosperm expression, zein metabolism regulation, flavonoid biosynthesis regulation, and cell cycle regulation.

As illustrated in **Table 2**, the promoters of most *GmFAD* genes contained cis-elements related to phytohormone responses, including ABRE, TGA, P-box, TATC-box, GARE-motif, CGTCA-motif, TGACG, AuxRR-core, and TGA-box, which correspond to ABA, SA, GA, MeJA, and Auxin signaling, respectively. Additionally, TC-rich repeats, which often function on the regulation of defense and stress responses, have been identified within the promoter sequences of 10 *GmFAD* genes (*GmFAD3.3*, *GmFAD7-2*, *GmFAD2.1*, *GmFAD2.2*, *GmFAD2.5*, *GmDES1.2*, *GmSLD1.1*, *GmSLD1.4*, *GmSLD1.5*, and *GmSLD1.6*). Moreover, the distribution patterns of TC-rich repeats within the *GmFAD2* subfamily were similar to those in the *GmSLD* subfamily, but it is notably absent in the *GmFAD6*, *GmFAD5*, and G*mFAB2* subfamilies.

**Table 2:** The main cis-elements in *GmFAD* gene promoter regions in soybean.

No.	Cis-elements	Function
1	Box 4, G-Box, GT1-motif, ACE, AE-box, AT1-motif, ATCT-motif, chs-CMA1a, GA-motif, Gap-box, GATA-motif, I-box, LAMP-element, MRE, TCCC-motif, TCT-motif, 3-AF1 binding site	Light responsive
2	ABRE	Abscisic acid responsiveness
3	TCA-element	Salicylic acid responsiveness
4	P-box, TATC-box, GARE-motif	Gibberellin-responsive
5	CGTCA-motif, TGACG-motif	MeJA-responsiveness
6	TGA-element, AuxRR-core, TGA-box	Auxin-responsive
7	TC-rich repeats	Defense and stress responsiveness
8	MBS	Drought-inducibility
9	circadian	Circadian control
10	ARE	Anaerobic induction
11	HD-Zip 1	Cell cycle regulation
12	LTR	Low-temperature responsiveness
13	CAT-box	Meristem expression
14	CCAAT-box	MYBHv1 binding site
15	O2-site	Zein metabolism regulation

All members of the *GmFAD* gene family possess abundant light-responsive cis-elements at the start codon upstream, with the detection of 17 such regulatory elements, such as Box4, G-Box, and GT1-Motif and others, suggesting that light signals play crucial roles in the accumulation of soybean fatty acid desaturases. Overall, these findings illuminate the potential functions of *GmFAD* genes in facilitating plant growth and development, enhancing stress responses, and modulating hormone signaling.

### Gene ontology (GO) functional annotation and KEGG pathway enrichment analysis of *GmFAD* genes

**Figure 6:**
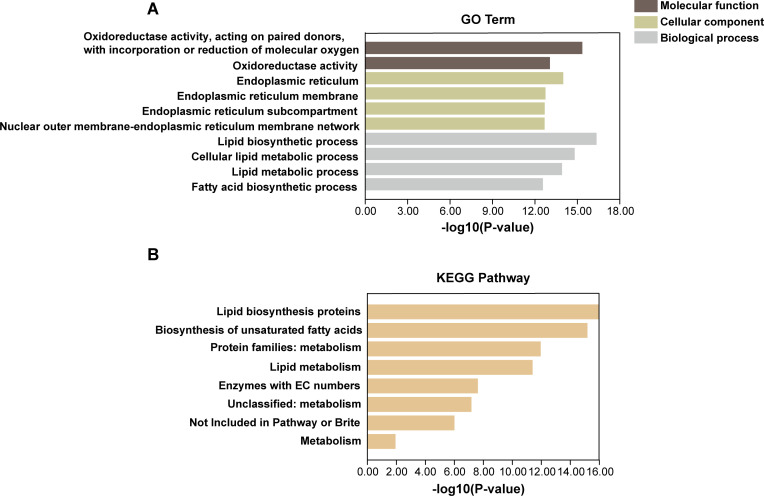
GO functional annotation and KEGG pathway enrichment analysis of *GmFAD* gene family. **(A)** Gene ontology (GO) analysis of the *GmFAD* gene family. **(B)** The KEGG pathway analysis of the *GmFAD* gene family. Both GO enrichment and KEGG pathway enrichment analysis were performed using TBtools, respectively. A total of 10 GO terms and 10 KEGG pathways with the lowest P-value were listed. The horizontal axis shows -log10(P-value), and the larger P-value means higher statistical significance.

To gain a deeper understanding of the functional pathways associated with *GmFAD* genes, we conducted Gene Ontology (GO) annotation and Kyoto Encyclopedia of Genes and Genomes (KEGG) pathway enrichment analysis to explore the potential functions of *GmFAD*. The results showed that *GmFAD* was mainly enriched in three GO terms: “molecular function”, “cellular component” and “biological process” (**Figure 6A**). Notably, the *GmFAD* gene family were significantly implicated in oxidoreductase activity, facilitating the transfer of electrons between paired donors and binding or reduction of molecular oxygen, and *GmFAD* genes also exhibited acyl-acyl-carrier-protein desaturase activity. Furthermore, *GmFAD* genes showed significant enrichment in membrane components, particularly endoplasmic reticulum membrane network, suggesting their potential involvement in the stability of membrane assembly. Additionally, both GO and KEGG annotations revealed that *GmFAD* genes were significantly involved in the biosynthetic and metabolic pathways of lipids and fatty acids (**Figure 6B**). In summary, *GmFAD* gene family participate in the biosynthesis and metabolism of fatty acid and lipid, and they also are responsible for redox and desaturation activities.

### Expression patterns of *GmFAD* genes in soybean after *F. fujikuroi* infection

**Figure 7:**
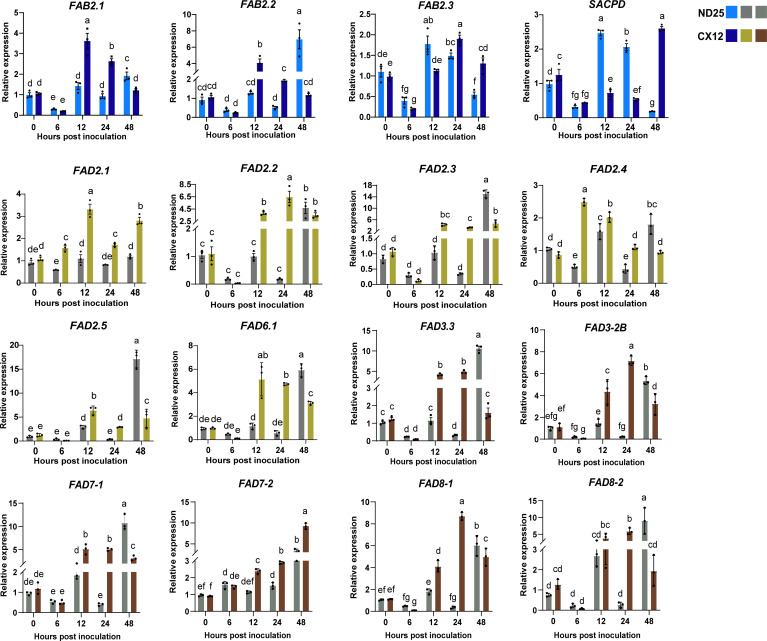
Expression patterns of *GmFAD* genes in soybean after *F. fujikuroi* inoculation. The expression level of *GmFADs* at 0 hour post inoculation was normalized as “1”. Quantitative RT-PCR was used to investigate the expression levels of each *GmFAD* gene upon *F. fujikuroi* infection from three biological and four technical replicates. Vertical bars indicated the standard errors of mean. Means denoted by the same lowercase letters when there was no significant difference at P < 0.05 as determined by Analysis of variance (ANOVA) and Duncan’s multiple range test (DMRT) using SPSS 24 software.

To investigate the regulation of *GmFAD* genes in soybean seed resistance to *F. fujikuroi*, the dominant seed decay pathogen, we analyzed expression patterns of 16 *GmFAD* genes in soybean seeds of susceptible cultivar (CX12) and resistant cultivar (ND25) after *F. fujikuroi* inoculation (**Figure 7**). As shown in **Figure 7**, all representative *GmFAD* genes belonging to ω-3 and -6 desaturase subfamilies were significantly induced by *F. fujikuroi* infection but their expression patterns differed in the resistant ND25 and susceptible CX12. Most genes in the susceptible CX12 were significantly up-regulated at much earlier infection (12 hpi), whereas they were strongly induced at 48 hpi in the resistant ND25. In particular, relative expression level of five genes including *GmFAD8.2*, *GmFAD3.3*, *GmFAD7-1*, *GmFAB2.2*, *GmFAD2.3* and *GmFAD2.5* were as higher as 6.0 fold at 48 hpi as compared to 0 hpi. In contrast, expression of *GmFAB2.3* and *GmSACPD* was inhibited at the early infection of *F. fujikuroi* (6 hpi) in both cultivars, but their expression were more rapidly recovered and reached the peak at 12 hpi in the resistant ND25 rather than those in the susceptible CX12. Additionally, *GmFAD2.4* were dramatically up-regulated by *F. fujikuroi* at 6 hpi and then decreased after 24 hpi in the susceptible CX12, but it had different expression pattern in the resistant ND25. Expression of *GmFAD2.1* and *GmFAD7.2* were weakly induced in the resistant ND25 upon *F. fujikuroi* infection but they were up-regulated differently in the susceptible CX12. This suggests that *GmFAD* genes play important roles in soybean seed resistance to *F. fujikuroi* infection.

## Discussion

In recent years, the *FADs* gene family has been extensively studied in a wide range of plant species, and genome-wide analyses have revealed different members of *FAD* genes regarding plant species (Zhiguo et al., 2019). Previously, Chi et al. (2011) reported 41 *GmFAD* genes from an older version of the Williams 82 genome. Zhang et al. (2021) identified 23 *GmFADs* in *Glycine max* var. Williams 82 using the BLASTP method when compared to Chinese wild soybean and ancient polyploid soybean. In this study, we identified 30 full-length *GmFADs* genes from the soybean genome (*Glycine max* Wm82.a4.v1) using a Hidden Markov Model (HMM) of protein structural domains by HMM BLAST software. The difference in total gene numbers of Williams 82 may result from different genomic versions and identification criteria and parameters of the BLAST software. Phylogenetic analysis based the amino acid showed 30 GmFAD proteins in this study were categorized into seven subfamilies, including FAD3/7/8, FAD2, FAD6, FAD5(ADS), DES, and SLD, and FAB2, and except for the ADS subfamily, other six subfamilies are basically consistent with those of *A. thaliana* and rice (Cheng et al., 2022), indicating that those subfamilies have a common ancestor before their divergence. For the ADS family, responsible for desaturating palmitic acid to palmitoleic acid (Laureano et al., 2021), *GmFAD5* was clustered with eight *AtADS* genes, whereas the monocotyledons rice lost this subfamily. Similar results was also reported in banana genome previously (Cheng et al., 2022), and this suggests that the *ADS* family might be formed after the differentiation of monocotyledons and dicotyledons. In addition, compared to *A. thaliana* and rice, the soybean genome possesses a larger number of genes within the ω-3 and ω-6 subfamilies as well as the SLD subfamily, and this can be predicted that these subfamilies have undergone positive selection pressures, leading to the expansion of gene families. This expansion may have granted soybean greater functional redundancy, enhancing their adaptability and diversity in lipid biosynthesis pathways. As a result, this could improve their capacity to adapt to environmental changes. Additionally, our results also demonstrated that different members within the same *GmFAD* gene subfamily were located on different soybean chromosomes, which is consistent with the previous findings in sunflower (Laureano et al., 2021).

Previous studies have revealed that expansion of the *FAD* gene family is species-specific in different plants, and this expansion is often determined by gene duplication events (Johnson and Thomas, 2007). Gene duplication plays a crucial role in generating new genes and functions, and both segmental and tandem duplication can drive the emergence of novel gene families (Cannon et al., 2004). In the wheat genome, *TaFAD* gene pairs were generated from tandem and segmental duplication with the pair number of 26 and 126, respectively (Hajiahmadi et al., 2020). In the poplar genome, *PtFAD* genes had 16 segmental repeat events and one tandem repeat event, respectively (Wei et al., 2022). However, replication of *FAD* family in *Brassica juncea* is mostly identified as segmental replications (89%) (Xue et al., 2020). In our study, there were 29 pairs of segmental repeats and one pair of tandem repeats in soybean *GmFAD* genes. Thus, segmental duplication in *FAD* family amplification is more frequently and important than tandem duplication, and it might play a vital role in increasing the genetic diversity of soybean *GmFAD* gene family. In addition, the selection pressure analysis showed that most *GmFAD* genes have undergone purifying selection to maintain the functions of *GmFAD* genes and species stability in soybean. For example, the *FAD2* gene has experienced several duplication events, and all members of the *FAD2* gene family have diverged into monopolistic and dicotyledons clusters in their evolutionary history. In some dicotyledons, the *FAD2* genes is divided into two branches as constitutive and specific expression. In soybean, multiple *GmFAD2* copies, such as *GmFAD2.1*, *GmFAD2.2*, *GmFAD2.3, GmFAD2.4*, and *GmFAD2.5*, have already been reported (Chi et al., 2011). Among them, *GmFAD2.2* and *GmFAD2.3* are constitutively expressed in both vegetative tissue and developing seeds, whereas two alleles *GmFAD2-1A* and *GmFAD2-1B* are specifically expressed in developing seeds and contributes to the polyunsaturated fatty acid contents of seed storage oil (Schlueter et al., 2007; Zhao et al., 2019). Another study on soybean genome analysis found that the transcript of *GmFAD2-2C* rather than *GmFAD2-2A* and *GmFAD2-2B* alleles of *GmFAD2-2* was significantly accumulated in pods under cool conditions (Li et al., 2007).

The variation in gene structure is critical for the functional evolution of gene family (Cao and Shi, 2012). With the exception of *GmFAD2.5* and *GmFAD6.2*, members within the same *GmFAD* subfamily exhibit similar intron/exon structures and intron patterns, and the proteins encoded by the same gene family had similar gene motif compositions and conserved protein domains. Similar results have also been found in *Medicago truncatula* (Zhang et al., 2018), wheat (Hajiahmadi et al., 2020), and *Brassica napus* (Xu et al., 2019). Our study also revealed that no common conserved sequences existed among these 30 *GmFAD* genes, and gene members clustered into the same clade were evolutionarily conserved. It is implied that specific conserved motifs play different roles in plant growth and development (Zhang et al., 2015). Combined with the results of the phylogenetic analyses, these results strongly support the reliability of the taxon divisions.

Previous studies have demonstrated that the *FAD* gene promoters contained diverse *cis*-elements in response to growth, development, fruiting, and defense and stresses (Soria-Garcï et al., 2019). Horiguchi et al. (1996) that the expression level of the wheat *TaFAD7* gene was significantly up-regulated during leaf development stage under light and dark stresses. Liu et al. (2016) have reported that the *BnFAD2*-C5 promoter had SA and JA response elements, and their expression was up-regulated by SA and JA induction. In this study, we identified a range of *cis*-elements in the promoter region of *GmFAD* genes related to low temperature, drought, light, hypoxia, circadian rhythms, plant hormones (ABA, GA, SA, MeJA), and defense and stress responsive. For example, TC-rich repeats involving in the regulation of defense and stress responses were identified in the promoter sequences of 10 *GmFAD* genes including *GmFAD3.3*, *GmFAD7-2*, *GmFAD2.5*, *GmFAD2.2*, *GmFAD2.1*, *GmDES1.2*, *GmSLD1.5*, *GmSLD1.4*, *GmSLD1.1* and *GmSLD1.6*, suggesting that these genes may play important roles in soybean defense and stress responses. However, expression analysis of Chinese wild soybean under salt stress demonstrated that *GsDES1.1*, *GsDES1.2*, *GsFAD2.1* and *GsSLD1* in leaves were not closely related to salt stress response (Zhang et al., 2021).

Growing evidence that members of the *FAD* gene family regulate plant defense responses to biotic stresses (Kachroo et al., 2005; Dar et al., 2017). Previous studies demonstrated *AtFAD2* genes not only contribute to salt and cold tolerance in *A. thaliana* (Schlueter et al., 2007; Chi et al., 2011; Zhao et al., 2019), but also some alleles of *GmFAD2* genes was able to respond to pathogen attack by increasing the biosynthesis of linoleic acid and palmitic-linoleic acid in soybean (Hernández et al., 2011). Li et al. (2021) showed that *HaFAD3.1* and *HaADS6* genes in sunflower were expressed at higher level after *Orobanche cumana* infection. In *Brassica carinata*, five genes including *BolFAD6.2*, *BolFAD4.3*, *BolFAD6.3*, *BolFAD4.1*, and *BolADS17*, were significantly up-regulated in response to *Xanthomonas campestris* infection (Shaheen et al., 2023). The *Arabidopsis* double mutant *fad7/fad8* also exhibited reduced the accumulation of triene fatty acid in chloroplast and increased the sensitivity to *Pseudomonas syringae* pv. tomato DC3000 (Yaeno et al., 2004). In addition, several genes such as *DES1*, *FAB2.3*, *FAB2.5*, *FAB2.7* and *FAD3.2* were significantly down-regulated in banana when infected by the pathogenic FocTR4 causing banana wilt disease (Cheng et al., 2022). FAD genes play important roles in membrane remodeling and signaling in grapevine defense towards biotrophic pathogens (Cavaco et al., 2021). During the early interaction between grapevine and the biotrophic oomycete *Plasmopara viticola*, the polyunsaturated alpha-linolenic acids were highly accumulated in the leaves of the tolerant genotype, followed by alterations in the expression of desaturase genes, regulation of membrane fluidity, and finally JA accumulation and activation of JA biosynthetic pathway, which forms together form a complex network of disease resistance responses in grapevines. Especially, the expression patterns of *FAD6* and *FAD8* may be related to the timing of the JA pathway activation during the interaction of grapes with the necrotrophic pathogen *B. cinerea* (Cavaco et al., 2021).

In Southwest China, soybeans are often exposed to heavy rainfall and high humidity from the full pod stage (R4) to the ripening stage (R8), resulting in severe pod rot and seed decay, and largely reduced soybean yield and quality (Chang et al., 2020a). In this study, the *GmFAD* gene was also significantly induced by *F. fujikuroi* causing soybean seed decay. Specially, eleven of the representative 16 *GmFAD* genes were significantly up-regulated at the early infection stage of *F. fujikuroi* in the susceptible cultivar (CX12) seeds, whereas no significant changes were observed in resistant cultivar (ND25) seeds until 48 hpi. Our study showed that at the late stage of *F. fujikuroi* infection, the genes *GmFAB2.1/2.2*, *GmFAD3.3*/*3-2B*/*7-1*/*8-2*, and *GmFAD2.3/2.5* were expressed significantly higher in resistant cultivar (ND25) than in sensitive cultivar (ST06). This suggests that some *GmFAD* genes are involved in pathogen defense responses. Since *F. fujikuroi* is one necrotrophic fungi, JA signaling can be activated as plant immunity (Song et al., 2023). In addition, *F. fujikuroi* is also well-known as the causal agent of rice bakanae bring big threat to rice production worldwide, and transcription profile of rice to *F. fujikuroi* infection found the pathways involved in bakanae resistance, such as chitin, JA-dependent signaling, and hypersensitive response (Matić et al., 2016). Furthermore, *OsWRKY114* has been reported to act as a player in rice JA-mediated immunity against *Fusarium fujikuroi* in rice (Song et al., 2023).Therefore, we hypothesized that there may be a more persistent stimulation of unsaturated fatty acid (UFA) biosynthesis in resistant cultivar (ND25) seeds, which could lead to the induction of the JA pathway (Cavaco et al., 2021). This is also consistent with the prediction of the promoter *cis*-acting elements of *GmFAD* genes in response to JA. In conclusion, *GmFAD* genes play crucial roles in soybean seed resistance against the seed decay fungus *F. fujikuroi*, and the diversity of enzyme functions and expression patterns in the *GmFAD* gene family suggests the diversity of gene functions (Laureano et al., 2021). The results will enhance our understanding of the regulatory genes involved in the biosynthesis pathway of unsaturated fatty acids. In the future, gene editing technology will enable precise regulation of *GmFAB2.1/2.2*, *GmFAD3.3/3-2B/7-1/8-2*, and *GmFAD2.3/2.5*, allowing for the breeding of soybean varieties with enhanced resistance and good agronomic traits.

## Conclusion

In this study, 30 full-length *GmFAD* genes were identified from the soybean genome. Analyses of gene structure, protein three-dimensional structure, and conserved motifs and conserved structural domains indicate that *GmFAD* genes are clustered into seven subfamily and evolutionarily conserved, and this is also strongly supported by the phylogenetic analysis. We found segmental duplication plays an important role in the gene amplification and subfamily generation of *GmFAD* in soybean genome. Most *GmFAD* gene promoter can be activated by light, phytohormones and other abiotic stresses, thus inducing fatty acid biosynthesis. Expression of *GmFAD* genes were differentially induced in the resistant and susceptible cultivars under seed decay stress caused by *F. fujikuroi*, some specific up-regulated genes such as *GmFAB2.1*/*2.2*, *GmFAD3.3*/*3-2B*/*7-1*//*8-2*, and *GmFAD2.3*/*2.5* would be the potential candidate genes for seed-decay resistance breeding in soybean. Future studies will explore the roles of these genes in seed decay stress. The results obtained are crucial for researching the molecular mechanisms of fatty acid synthesis, *FAD* and *SAD* editing, and for marker-assisted and genomic selection in breeding soybean varieties with a specific fatty acid composition in their oil.

## Materials and methods

### Identification and physicochemical characterization of soybean *FAD* gene family

The genome data of *Glycine max* var. Williams 82 were retrieved from the Genome Warehouse (GWH) with the Phytozome v13 database (Annotation version: *Glycine max Wm82.a4.v1*, https://phytozome-next.jgi.doe.gov/) (Xu et al., 2019). The published protein sequences and gene sequences of 24 *AtFADs* were obtained from TAIR release 10 (http://www.arabidopsis.org) (Shaheen et al., 2023), while the published sequences of 18 *OsFADs* were downloaded from RGAP release 7 (http://rice.plantbiology.msu.edu) (Zhiguo et al., 2019).

The hidden Markov model (HMM) profiles for the FA_desaturase (PF00487), FA_desaturase 2 (PF03405), and TMEM189 (PF10520) domains (Cheng et al., 2022) downloaded from the Pfam protein family database (https://www.ebi.ac.uk/interpro/entry/pfam/) were searched against the soybean (*Glycine max* var. Williams 82) protein data using an e-value threshold of ≤1e ^-5^. The *GmFAD* gene family members were then filtered to eliminate duplicates and identify potential gene family members. Subsequently, the protein physicochemical properties and subcellular localization of the *GmFAD* members were further analyzed using Expasy (https://www.expasy.org/) and Cell-PLoc (http://www.csbio.sjtu.edu.cn/bioinf/Cell-PLoc-2/).

### Evolution analysis of *FAD* gene family in soybean

We performed multiple alignments of *FADs* were conducted by Clustal W using full-length protein sequences from soybean (30), *A. thaliana* (24), and rice (18), respectively. A Neighbor-Joining (NJ) phylogenetic tree was constructed using the p-distance model by MEGA7 (http://www.megasoftware.net) (Kumar et al., 2016). The bootstrap support values were calculated from 1000 repeats. Subsequently, the evolutionary tree was refined and visualized through the iTOL online platform (https://itol.embl.de/).

### Comprehensive analysis of chromosome locations and gene duplications

The annotation file for the general feature format version 3 (GFF3) in the soybean genome database was utilized to pinpoint the chromosome locations of the *GmFAD* genes. Visualization of the chromosomal localization of the *GmFAD* genes were achieved using TBtools software, which was based on the starting position on the soybean chromosome (Chen et al., 2020). The matched sequences covered more than 80% of the length of the longer gene, exhibited over 80% similarity within their respective regions, and were products of a single duplication event (Jiang et al., 2013; Singh and Jain, 2015). To further assess the evolutionary pressure on the *GmFAD* gene family, the synonymous (*Ks*) and non-synonymous (*Ka*) substitution rates of the *GmFAD* gene pairs were computed using TBtools, along with the *Ka/Ks* calculator (Bailey et al., 2006; Kong et al., 2013; Kumar et al., 2016). In addition, to explore the evolutionary relationships within the soybean species, MCScanX and BLASTP were employed to detect gene pairs that were co-variantly associated with *FAD* members (Xue et al., 2020).

### Investigation of gene structures, conserved structural domains, conserved motifs and protein structures of *GmFADs*

The soybean coding sequence and genome file were applied to explore the splicing phase of the *GmFADs* family (Zhang et al., 2021). The Gene Structure Display Server (GSDS, http://gsds.cbi.pku.edu.cn/).and TBtools software (Chen et al., 2020) were employed to map the distribution of introns, exons and non-coding regions within genes. NCBI Conserved Domains database (http://www.ncbi.nlm.nih.gov/Structure/cdd/wrpsb.cgi) was used to identify the conserved structural domains of *GmFAD* gene family proteins, and MEME (https://meme-suite.org/meme/) to analyze the conserved motifs of *FAD* gene family proteins with the following parameter settings: the motif discovery was set to classical mode with a predicted motif count of 20, and each motif was allowed to occur 0 or 1 times (Bailey et al., 2006). The Pfam database was then utilized to evaluate the functions of the aforementioned motifs (Mistry et al., 2021). The conserved motifs and structural domains of the *FAD* gene family proteins were simultaneously visualized, and their interactions were analyzed using TBtools. The integrated structure of *GmFAD* was constructed using the Swiss-Model platform (https://swissmodel.expasy.org/) based on fragments of iterative templates (Wei et al., 2022), and subsequently refined and visualized by Chimera software to yield a three-dimensional structural model.

### Promoter analysis, GO annotation and KEGG enrichment analysis

Sequences encompassing the 1500 bp region upstream of the start codon (ATG) for each *GmFAD* gene were retrieved from the soybean genome database using TBtools. Subsequently, the promoter sequences of these Gm*FAD* genes were uploaded to Plant CARE (https://bioinformatics.psb.ugent.be/webtools/plantcare/html/) to identify cis-regulatory elements (Lescot et al., 2002).

The functional annotation of genes was conducted using the eggNOG-mapper database (http://eggnog-mapper.embl.de/). To further understand the potential pathways that might be associated with the *GmFAD* genes, the TBtools eggNOG-mapper Helper tool was subsequently employed to systematically organize and process the results derived from the eggNOG-mapper. Following this, the GO-basic file and the KEGG-backend file were exported for GO enrichment analysis and KEGG Pathway enrichment, respectively (Wei et al., 2022).

### Plant materials, growth conditions and pathogen inoculation

The fungal isolate *F. fujikuroi* (No. S100) was isolated from the rot seeds of soybean in the fields and identified using sequence analysis of *translation elongation factor 1 alpha* (*EF-1α*) and DNA-directed RNA ploymerase II second largest subunit (*RPB2*) (Chang et al., 2020a). The cultivar CX12 exhibited susceptibility to *F. fujikuroi*, whereas the cultivar ND25 showed a high resistance to the fungus, and both these two cultivars were chosen to examine the expression of *GmFAD* genes following inoculation of soybean seeds with *F. fujikuroi*.

Spore suspensions of *F. fujikuroi* were prepared following the protocol by Chang et al. (2022). For sporulation, a mung bean liquid medium was prepared by boiling 30 g of mung bean in 1 L of sterilized water for 20 min, filtering the mixture with cheesecloth, and then autoclaving at 121℃ for 30 min (Lv and Sun, 2007). Disease-resistant and susceptible soybean seeds were inoculated with *F. fujikuroi* suspensions at a concentration of 1 × 10^6^ spores per milliliter, supplemented with 0.1% Tween 20. As a control (CK), soybean seeds were treated with an equal volume of mung bean liquid medium. The treated and control seeds were then arranged on water agar medium (WA), and were incubated in the dark at a constant temperature of 25 ℃ for varying durations of 0, 6, 12, 24, and 48 h, respectively. The experiments were conducted in triplicate. At specified intervals post inoculation, soybean seeds from both control and treatment groups were collected, immediately frozen in liquid nitrogen, and subsequently stored at -80°C for RNA extraction.

### Expression analysis of *GmFAD* genes in soybean seeds upon *Fusarium fujikuroi* infection

The expression profiles of twenty-six *GmFAD* genes were analyzed using qRT-PCR with specific primer pairs listed in **Supplementary Table S6**. Total RNA was extracted from soybean seed samples using Fast Pure^®^ Universal Plant Total RNA Isolation Kit (Vazyme-Bio, Chengdu, China) and subsequently assessed with a NanoDrop fluorometer (Thermo Fisher Scientific, Stuttgart, Germany) to ascertain the concentration and quality of RNA. First-strand cDNA was synthesized from 2.5 μg RNA in a 20-μL reaction volume according to the instructions of BeyoRT™II First Strand cDNA Synthesis Kit (Beyotime-Bio, Shanghai, China). The qRT-PCR assay was conducted on the Chromo4 Real-Time PCR System (Bio-Rad, CA, USA), within a 10-μL reaction mixture containing 5 μL of the SYBR qPCR Mix (2×) (Vazyme-Bio, Shanghai, China), 1 μL of each primer (10 mM), 1 μL of template DNA (10 ng), and 2 μL of ddH_2_O. The PCR thermal cycling conditions were set as follows: an initial heat activation at 95°C for 3 minutes, followed by 40 cycles of amplification at 10 s at 95°C for 10 s, annealing temperature for 30 s, 15 s at 95°C, 1 min at 60°C, and a final 15 s at 95°C for melting curve analysis. Relative gene expression levels were determined using the 2^-ΔΔCt^ method (Livak and Schmittgen, 2001) with the *GmActin* gene serving as an internal reference for normalization. Each sample was subjected to four technical replicates and three biological replicates. Data analysis was preformed using SPSS 24 software (SPSS Software Inc., Chicago, IL, USA), and statistical analysis was conducted with Analysis of variance (ANOVA) and Duncan’s multiple range test (DMRT). The results were visualized with GraphPad Prism 10 (GraphPad Software Inc., San Diego, CA, USA).

## Data availability statement

All data generated or analyzed during this study are included in this published article and its supplementary information files. The general feature format (GFF) sequence file and the protein sequence file of soybean (*Glycine max* var. Williams 82) used in this study are available at Phytozome v13 database (https://phytozome-next.jgi.doe.gov/). The sequences used for the interspecific covariance homology relationships between soybean, rice and *Arabidopsis*, corresponding FAD sequence information are available in the rice database (http://rice.plantbiology.msu.edu) and TAIR database (https://www.arabidopsis.org/), respectively.
